# Bedside Intraoral Scanning to Reduce Procedural Burden in Older Adults With Dementia: Nonrandomized Counterbalanced Crossover Feasibility Study

**DOI:** 10.2196/92879

**Published:** 2026-08-06

**Authors:** Anna-Lena Hillebrecht, Kirstin Vach, Christoph Maurer, Ralf J Kohal, Benedikt C Spies

**Affiliations:** 1 Department of Prosthetic Dentistry, Center for Dental Medicine, Medical Center ‑ University of Freiburg, Faculty of Medicine, University of Freiburg, Hugstetter Straße 55, Freiburg, 79106, Germany, 49 76127048440; 2 Institute of Medical Biometry and Statistics, Faculty of Medicine, Medical Center - University of Freiburg, Freiburg, Germany; 3 Center for Data Science, Eberswalde University for Sustainable Development, Eberswalde, Germany; 4 Department of Neurology and Neurophysiology, University Medical Center Freiburg, Freiburg, Germany

**Keywords:** dementia, aged, geriatric dentistry, intraoral scanning, digital health, feasibility studies, patient-reported outcome measures, procedural burden

## Abstract

**Background:**

Older adults living with dementia often have limited tolerance for intraoral procedures, which can restrict access to even basic oral health assessment in clinical care. Procedural burden—characterized by sustained intraoral manipulation, sensory overload, and limited opportunities for interruption—may represent a key barrier to equitable oral care. Intraoral scanning (IOS) enables stepwise, interruptible acquisition of oral data and may reduce this burden, but feasibility and tolerability in people living with dementia have not been systematically evaluated.

**Objective:**

This study aimed to assess the feasibility and tolerability of bedside maxillary IOS compared with a conventional impression procedure in people living with dementia.

**Methods:**

A single-center, nonrandomized, counterbalanced, crossover feasibility study was conducted in a geriatric ward. Nineteen hospitalized participants with dementia (mean age 83.5, SD 3.4 years; mean Mini-Mental State Examination score 18.2, SD 4.8) underwent both bedside maxillary IOS and a conventional alginate-based impression procedure during a single visit. The order of procedures was not randomized but assigned using an alternating allocation sequence based on chronological enrollment to counterbalance potential order effects. The primary outcome was feasibility, defined as procedure completion without premature termination due to gag reflex, defensive movements, participant refusal, or withdrawal of assent or cooperation. Secondary outcomes included participant-rated discomfort using a 0 to 10 visual analog scale (VAS); clinician-rated manageability using a 0 to 10 VAS, with higher scores indicating greater difficulty; adverse reactions, including gag reflex and defensive movements; and active bedside procedural time in minutes. Paired comparisons were performed using the Wilcoxon signed-rank tests for VAS outcomes, a sign test for active bedside procedural time, and McNemar tests for binary outcomes, with a 2-sided significance level of .05.

**Results:**

Participants reported significantly lower discomfort with IOS than with the conventional impression procedure (median 1.0, IQR 0.5-1.5 vs median 7.75, IQR 3.7-8.5; *P*<.001), and clinician-rated manageability was more favorable for IOS (median 2.0, IQR 1.0-3.0 vs median 5.0, IQR 3.0-6.5; *P*<.001). Active bedside procedural time was similar in the analysis of all initiated procedures, with a mean duration of 3.02 (SD 0.72) minutes for IOS and 2.92 (SD 0.78) minutes for the conventional impression procedure. Exploratory analyses showed no statistically detectable evidence of an order effect. Gag reflex occurred less frequently during IOS (1/19, 5.3% vs 9/19, 47.4%; *P*=.004).

**Conclusions:**

Bedside IOS was feasible in hospitalized older adults living with dementia and was associated with lower immediate procedural burden than a conventional alginate-based impression procedure. These findings support further evaluation of IOS as a low-burden approach for oral data acquisition in dementia care, while downstream treatment effects and implementation in other settings require further study.

## Introduction

People living with dementia represent a growing and particularly vulnerable group with substantial unmet oral health needs [1-4]. Poor oral health in people living with dementia is associated with pain, impaired mastication, malnutrition, social withdrawal, and increased systemic risks, with the highest burden observed among care-dependent individuals [5-14].

Providing dental care for people living with dementia is frequently complicated by fluctuating cooperation, communication difficulties, behavioral symptoms, reduced tolerance to sensory stimuli, and limited ability to understand or endure sustained intraoral manipulation [15,16]. These challenges are particularly relevant in hospital or bedside settings, where patients may be medically frail, cognitively impaired, and unable to tolerate prolonged or continuous dental procedures. As a result, even basic intraoral assessment or prosthodontic procedures may become burdensome, delayed, incomplete, or omitted [17]. Conventional impression procedures can be especially challenging in this context because they require tray insertion, sustained mouth opening, tolerance of intraoral material during setting, and continuous cooperation until removal [18,19].

Intraoral scanning (IOS) has emerged as a digital alternative to conventional impressions and offers several potential advantages, including improved comfort, reduced gag reflex, shorter or comparable procedure times, and the ability to interrupt and resume data acquisition according to patient tolerance [20,21]. These characteristics may be particularly relevant for people living with dementia, not primarily because of the technological novelty of IOS, but because the procedure can be performed stepwise, adapted to the patient’s momentary cooperation, and interrupted when signs of distress occur. In patients with reduced cooperation or limited tolerance for intraoral procedures, the ability to pause, redirect, and resume may represent a clinically meaningful advantage over procedures that require continuous completion once initiated. Recent work in nursing home residents has shown that intraoral 3D scans can be used for diagnostic evaluations in long-term care settings, supporting the potential relevance of scan-based oral assessment in older and care-dependent populations [22]. Evidence from pediatric teledentistry and special care dentistry further suggests that digital intraoral data acquisition may support documentation, remote assessment, and care planning in selected populations [23,24]. More broadly, teledentistry has been discussed as a strategy to improve access to oral health care, although evidence quality, implementation context, and patient selection remain important considerations [25]. However, evidence from these populations cannot be directly transferred to hospitalized people living with dementia, whose ability to cooperate may fluctuate within minutes and whose distress responses may require immediate procedural adaptation. Therefore, the central question in dementia care is not whether IOS is generally more comfortable than conventional impressions, but whether it is feasible and tolerable under real-world bedside conditions in patients with cognitive impairment and reduced procedural tolerance. Within the broader field of digital dentistry, intraoral scanners represent one component of digital technologies used to digitize oral structures and support documentation, diagnosis, treatment planning, and communication [26-29]. According to the FDI Policy Statement on Digital Dentistry, the overarching aim of such technologies is to improve the quality, safety, and efficiency of oral health care [30]. However, in the context of dementia care, the relevant question is not digitalization per se, but whether digital intraoral data acquisition can be performed with lower procedural burden in patients with reduced cooperation and limited tolerance for sustained intraoral manipulation.

Despite increasing interest in digital dentistry, systematic data on the feasibility and acceptance of IOS in people living with dementia remain scarce. This evidence gap is partly attributable to the frequent exclusion of individuals with dementia from dental research, despite international frameworks emphasizing inclusion, participation, and equity in health care and research [31]. Early feasibility studies have shown that even highly vulnerable populations can be successfully recruited and retained in dental research, including periodontal interventions in individuals with mild dementia [32] and chairside oral prophylaxis in people with profound intellectual and multiple disabilities [33]. At the same time, geriatric dentistry is undergoing a conceptual shift toward holistic, function-oriented, and patient-centered care models that emphasize minimal intervention and interdisciplinary collaboration [34]. Within this framework, feasibility and procedural tolerability are prerequisites for equitable access to oral assessment and care. This is also consistent with recent evidence suggesting that oral health interventions may have potential relevance in dementia care, while the available clinical evidence remains limited and further studies are needed [35].

Therefore, this study aimed to compare the feasibility and tolerability of bedside digital IOS with a conventional maxillary impression procedure in people living with dementia using a nonrandomized counterbalanced crossover design. We hypothesized that IOS would (1) demonstrate comparable feasibility, (2) reduce participant-reported discomfort and adverse reactions, and (3) not prolong active bedside procedural time compared with conventional alginate impressions.

## Methods

### Study Design and Setting

**Figure 1.**
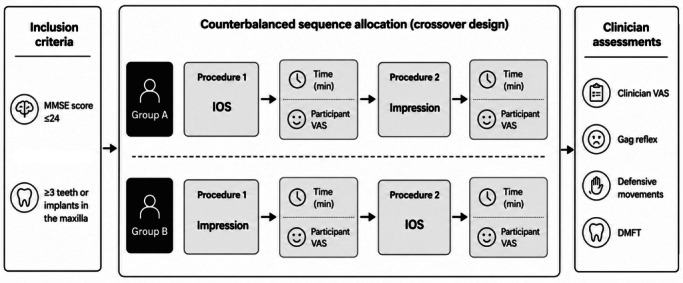
Study design of the nonrandomized counterbalanced crossover feasibility study. Areas highlighted in gray indicate the direct involvement of participants in the assessment process. Participants meeting the inclusion criteria (Mini-Mental State Examination [MMSE] score ≤24; ≥3 teeth or implants in the maxilla) were assigned by alternating allocation based on chronological enrollment to group A (first intraoral scanning [IOS], then conventional impression) or group B (first conventional impression, then IOS). For each procedure, active bedside procedural time and participant-rated visual analog scale (VAS) scores were recorded. Clinician assessments included VAS ratings, gag reflex, defensive movements, and decayed, missing, and filled teeth (DMFT) scores.

A single-center, nonrandomized counterbalanced crossover feasibility study was conducted on a geriatric ward of a university medical center in Germany. The design was therefore not a randomized crossover trial; sequence allocation was based on alternating chronological enrollment and was used only to counterbalance potential order-related effects. The study compared bedside maxillary IOS with a conventional impression procedure performed during the same visit. The investigation focused on feasibility and immediate procedure-related burden as prerequisites for low-burden oral data acquisition in people living with dementia, rather than on downstream treatment outcomes or implementation effectiveness. The study design, allocation procedure, assessment time points, and recorded outcomes are summarized in Figure 1.

### Ethical Considerations

The protocol was approved by the ethics committee of the University of Freiburg (EK 21-1525) and prospectively registered in the German Clinical Trials Register (DRKS00027119). The manuscript follows the CONSORT (Consolidated Standards of Reporting Trials) 2010 extension for pilot and feasibility trials where applicable. Consent was obtained from the legal representative, and participant assent was obtained immediately before each procedure.

### Participants and Recruitment

Eligible participants were adults with a clinical diagnosis of dementia and a Mini-Mental State Examination (MMSE) score of 24 or less. Inclusion criteria were at least 3 teeth or implants in the maxilla, ability to sit upright, and sufficient medical stability to tolerate a bedside dental procedure of up to 20 minutes. Exclusion criteria were lack of legal representative consent or participant assent, acute delirium, uncontrolled systemic disease, known allergy to latex or alginate, and maxillofacial conditions precluding impression taking or IOS.

Participants were identified in collaboration with ward staff. Study information was provided in easy-to-read German and supported by pictorial aids codeveloped with people living with dementia and the institutional accessibility office (Multimedia Appendix 1).

### Allocation and Sequence (Counterbalancing)

Participants underwent both procedures during a single visit. The purpose of allocation was to counterbalance potential order-related effects, such as fatigue, habituation, or increasing distress during prolonged bedside exposure. The order of procedures (IOS first or conventional impression first) followed an alternating allocation sequence based on chronological enrollment, aiming to achieve balanced sequence groups across the recruitment period. This allocation approach was chosen for pragmatic counterbalancing and was not intended to generate a random sequence. No computerized randomization or allocation concealment was performed; therefore, the design should be interpreted as a nonrandomized counterbalanced crossover study. Masking of participants and operators was not feasible due to the nature of the interventions. Data analysis was performed blinded to the intervention sequence where possible.

### Procedures and Interventions

Each participant underwent both a digital IOS and a conventional alginate impression during a single bedside session, according to the assigned counterbalanced sequence. Before the procedures, a brief dental examination was performed to record dentition status, including the number of teeth and the decayed, missing, and filled teeth (DMFT) index. Both procedures were performed at the participant’s bedside using dementia-sensitive communication, continuous monitoring of distress, and the predefined stopping criteria described below.

### IOS

Maxillary scans were acquired using a TRIOS 4 wireless intraoral scanner (3Shape) with single-use scanner tips. A standardized scan strategy was applied: occlusal surfaces were scanned from the right to the left posterior region, followed by the palatal aspect, and finally the buccal and labial surfaces from right to left. The procedure was performed stepwise and could be paused if signs of distress, refusal, or fatigue occurred. IOS was considered completed when a clinically usable maxillary scan had been acquired.

### Conventional Impression Procedure

A conventional maxillary impression was taken using nonperforated stock trays in sizes S, M, or L and an ISO 21563-certified alginate material (HS-Maxima Alginate, Henry Schein Inc). The tray size was selected clinically and tried in before impression taking. Alginate was hand-mixed according to the manufacturer’s instructions, with a mixing time of 30 seconds, working time of 90 seconds, and intraoral setting time of 90 seconds. The loaded tray was inserted intraorally, held in place during setting, and removed after completion of the setting time. A dental assistant provided bedside support and hand-mixed the alginate.

### Timing, Comparability, and Monitoring Between Procedures

Procedure duration was recorded as active bedside procedural time using a stopwatch. Active bedside procedural time was defined as the time during which the participant was directly involved in the procedure. For IOS, timing started with the first intraoral insertion of the scanner and ended when the maxillary scan was clinically completed. Scanner setup steps outside direct participant involvement, including case creation, entry of identifiers, scanner calibration, and data saving, were not included. For the conventional impression procedure, timing included tray selection and try-in, alginate mixing and loading, tray insertion, intraoral setting, and tray removal, because these steps were performed at the bedside and required participant presence and cooperation. Thus, the timing metric was intended to compare patient-facing procedural burden rather than the total digital or conventional workflow time.

No fixed washout period was prespecified between procedures. This pragmatic same-visit approach was chosen to minimize repeated patient transfers, additional consent or assent procedures, and disruption of routine geriatric ward care in a frail inpatient dementia population. It also reflected the study aim of comparing immediate patient-facing procedural burden under clinically realistic bedside conditions. After completion or termination of the first procedure, participants were offered a pause before the second procedure was initiated. The second procedure was started only if the participant appeared calm, was able to remain seated upright, and provided renewed assent. Verbal and behavioral signs of distress, refusal, fatigue, defensive movements, or increasing agitation were monitored continuously before and during both procedures. Because both procedures were performed within one visit, carryover effects due to fatigue, sensitization, or habituation cannot be fully excluded despite counterbalancing of the sequence.

### Dementia-Sensitive Conduct, Distress Management, and Stopping Criteria

Given the vulnerability of the study population, procedures were conducted using dementia-sensitive communication strategies and continuous monitoring of distress. Participants were informed that they could pause or stop at any time without consequences for their care. The operator monitored verbal and behavioral signs of refusal or distress throughout.

Predefined criteria for terminating a procedure were inability to proceed safely due to (1) gag reflex preventing continuation, (2) defensive movements compromising safety or feasibility, or (3) participant refusal or withdrawal of assent or cooperation.

### Operator Experience and Bedside Support

All procedures were performed by a single prosthodontist with board certification in geriatric dentistry and extensive experience in IOS (more than 200 cases) and conventional alginate impressions (more than 500 cases). The use of a single experienced operator was chosen to ensure consistent dementia-sensitive conduct and standardized application of both procedures in this feasibility study. However, this design does not allow separation of procedure-related effects from operator-related factors and may overestimate feasibility compared with settings involving less experienced operators.

A short run-in phase with 5 pilot patients was conducted to standardize procedure timing and administration of the visual analog scale (VAS); pilot cases were excluded from analysis.

### Outcomes

#### Primary Outcome

The primary outcome was feasibility, defined as procedure completion without premature termination due to gag reflex, defensive movements, participant refusal, or withdrawal of assent or cooperation.

#### Secondary Outcomes

Secondary outcomes were designed to capture procedure-related burden and clinical practicality. Participant-rated discomfort was assessed immediately after each procedure using a VAS ranging from 0 (no discomfort) to 10 (worst possible discomfort), supported by pictorial anchors. Clinician-rated manageability was assessed immediately after each procedure using a 0 to 10 VAS, with higher scores indicating greater difficulty to perform under routine conditions, considering patient tolerance and cooperation. Because the operator could not be blinded to the procedure, clinician-rated manageability was interpreted as a pragmatic implementation-related outcome rather than as a blinded objective end point. Adverse reactions were recorded as present or absent for gag reflex and defensive movements. Active bedside procedural time was recorded in minutes.

### Exploratory Analyses

Exploratory analyses assessed associations of outcomes with cognitive status (MMSE), dentition status (number of teeth and DMFT), and procedure order.

### Statistical Analysis

No formal sample size calculation was performed because of the feasibility design. Descriptive statistics were reported as means with SDs, medians, ranges, and frequencies with percentages, as appropriate. Paired comparisons between IOS and conventional impression taking were performed using the Wilcoxon signed-rank test for VAS outcomes, the sign test for active bedside procedural time, and McNemar test for binary outcomes. Between-group comparisons according to procedural sequence were conducted using rank-sum tests for continuous outcomes and the Fisher exact test for categorical outcomes. Associations between MMSE scores and oral health indicators were assessed using Spearman rank correlations. Agreement between participant-rated discomfort and clinician-rated manageability was evaluated using intraclass correlation coefficients (ICCs). All tests were 2-sided, with a significance level of .05. Analyses were performed using Stata (version 17; StataCorp LLC).

## Results

### Participant Flow and Baseline Characteristics

**Figure 2.**
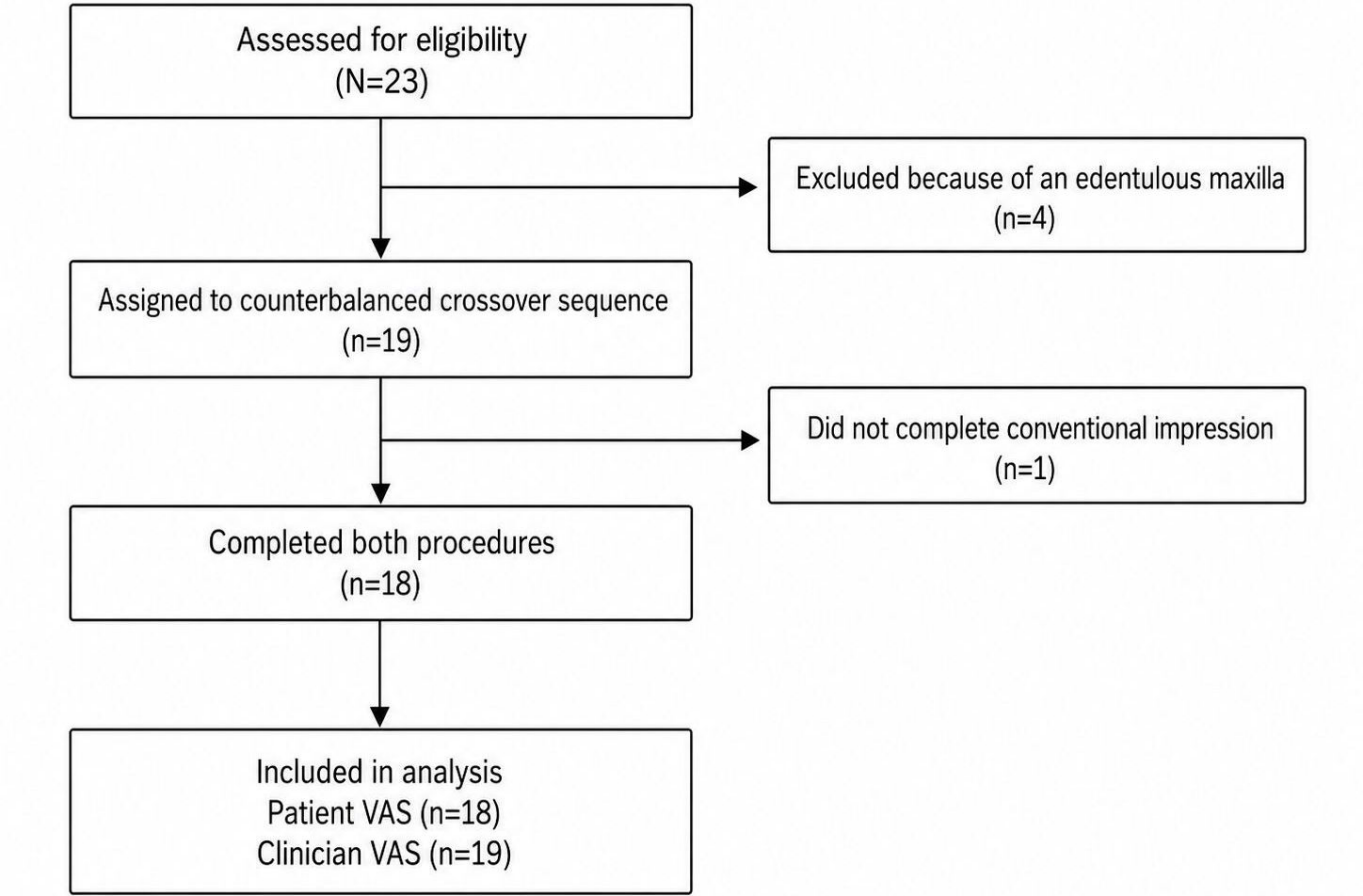
Participant flow diagram of the nonrandomized counterbalanced crossover feasibility study. Of the 23 individuals assessed for eligibility, 4 were excluded because of an edentulous maxilla. Nineteen participants were assigned by alternating allocation to a counterbalanced crossover sequence and received both interventions (intraoral scanning and conventional alginate impressions). A total of 18 participants completed both procedures; 1 conventional alginate impression was not completed because of participant noncooperation. The analysis included feasibility and adverse events (n=19), participant-rated visual analog scale (VAS) scores (n=18; 1 missing), and clinician-rated VAS scores (n=19).

Between November 2023 and June 2024, a total of 23 hospitalized patients with dementia were screened on the geriatric ward; 4 (17.4%) were excluded due to an edentulous maxilla. Nineteen (82.6%) participants were enrolled and underwent both procedures during a single bedside visit (Figure 2). Completion of the conventional impression procedure was prematurely terminated in 1 (5.3%) participant due to withdrawal of cooperation; IOS was completed in all participants.

The final sample comprised 19 participants with a mean age of 83.5 years (SD 3.4; range 78‐90 y) and a mean MMSE score of 18.2 (SD 4.8). Participants showed substantial oral treatment need, with a mean DMFT index of 22.1 (SD 8.1) and a mean of 17.2 (SD 10.1) teeth present. Procedure order was balanced, with 10 (52.6%) participants undergoing IOS first and 9 (47.4%) undergoing the conventional impression procedure first. Detailed baseline characteristics and procedure sequence are presented in Table 1.

**Table 1.** Baseline characteristics and procedure sequence of the study participants (n=19).

Characteristic	Value
Age (y), mean (SD; range)	83.5 (3.4; 78-90)
Women, n (%)	11 (57.9)
Men, n (%)	8 (42.1)
MMSE^a^ score, mean (SD)	18.2 (4.8)
Number of teeth present, mean (SD)	17.2 (10.1)
DMFT^b^ index, mean (SD)	22.1 (8.1)
Removable prosthesis present, n (%)	7 (36.8)
Intraoral scanning performed first, n (%)	10 (52.6)
Conventional impression performed first, n (%)	9 (47.4)

a MMSE: Mini-Mental State Examination.

b DMFT: decayed, missing, and filled teeth.

One (5.3%) participant did not provide a discomfort rating (participant VAS) after both procedures; therefore, participant-reported outcomes were analyzed using the available cases (n=18, 94.7%), whereas clinician ratings and procedural outcomes were analyzed for all participants (n=19, 100%).

### Primary Outcome: Feasibility (Completion)

Feasibility, defined as procedure completion without premature termination due to gag reflex, defensive movements, participant refusal, or withdrawal of assent or cooperation, was high for both procedures. IOS was feasible in all (19/19, 100%; 95% CI 82.4%‐100%) the participants. The conventional impression procedure was feasible in 18 (94.7%; 95% CI 74.0%‐99.9%) participants. The paired comparison did not indicate a statistically significant difference (McNemar test, *P*>.99). The only failure occurred during the conventional procedure due to withdrawal of cooperation; no IOS procedures required termination.

### Secondary Outcomes: Participant Discomfort and Clinician Manageability

#### Participant-Rated Discomfort

**Figure 3.**
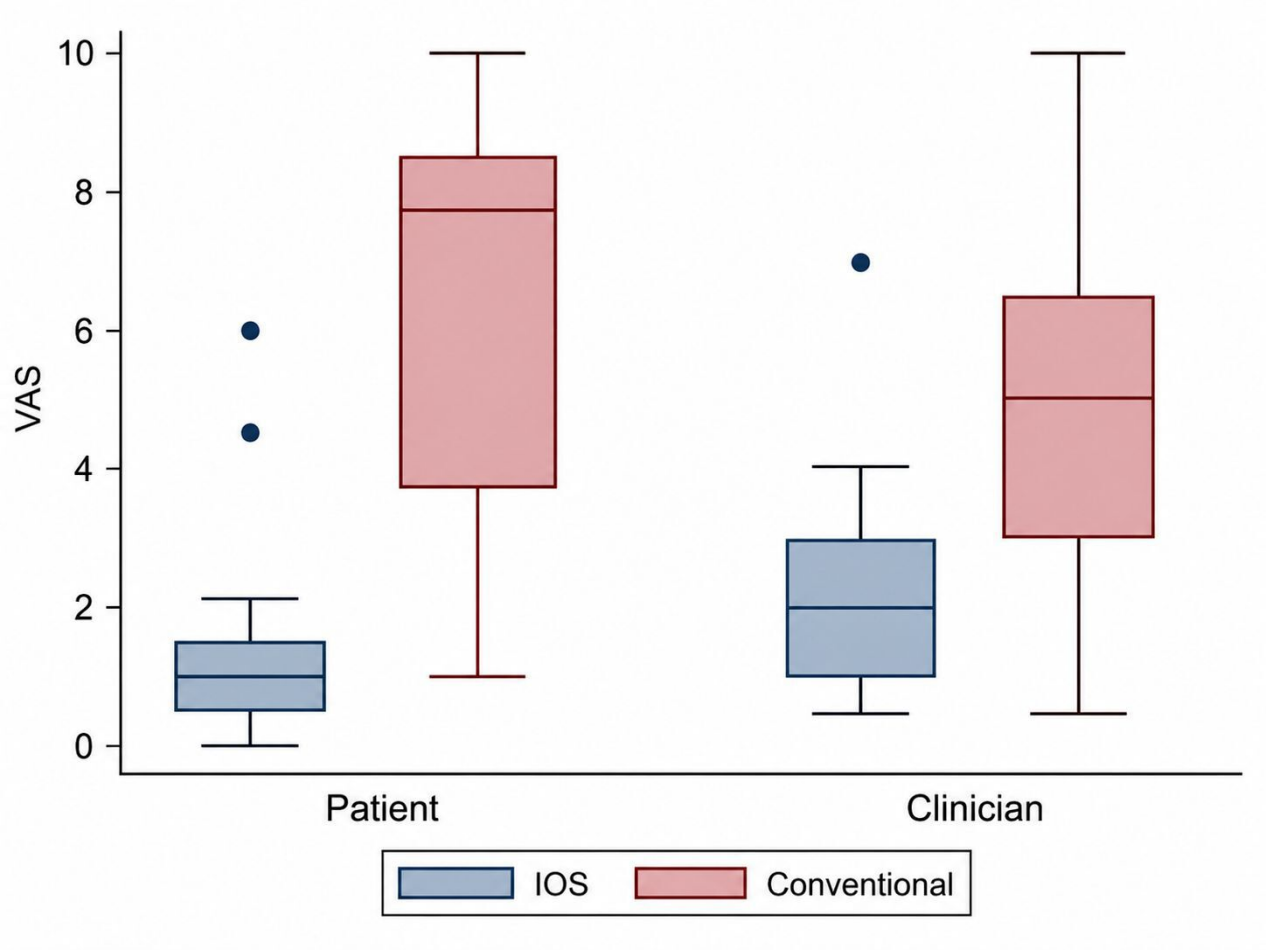
Visual analog scale (VAS) ratings of intraoral scanning (IOS) and conventional alginate impression taking by participants and the clinician. Participant-rated boxplots represent 18 available cases because 1 participant did not provide VAS ratings; clinician-rated boxplots represent all 19 participants.

Participants reported substantially lower discomfort for IOS than for the conventional procedure. Median discomfort was 1.0 (IQR 0.5-1.5) for IOS versus 7.75 (IQR 3.7-8.5) for the conventional procedure; corresponding mean values were 1.39 (SD 1.56) and 6.29 (SD 2.77), respectively (Wilcoxon signed-rank test, *P*<.001; n=18). The distribution of discomfort ratings was narrower for IOS, indicating more consistent tolerability across participants (Figure 3).

#### Clinician-Rated Manageability

The clinician rated IOS as more manageable than the conventional procedure. Median manageability scores were 2.0 (IQR 1.0-3.0) for IOS and 5.0 (IQR 3.0-6.5) for the conventional procedure; corresponding mean values were 2.29 (SD 1.54) and 5.02 (SD 2.52), respectively (Wilcoxon signed-rank test, *P*<.001; n=19). Participant and clinician ratings showed low agreement within each procedure (IOS: ICC 0.178; conventional impression procedure: ICC 0.180).

### Secondary Outcomes: Adverse Reactions

Among the 19 IOS procedures, gag reflex occurred in 1 (5.3%; 95% CI 0.1%‐26.0%) and in 9 (47.4%; 95% CI 24.4%‐71.1%) of the 19 conventional impression procedures, representing a significantly lower rate during IOS (McNemar test, *P*=.004). Defensive movements occurred in 3 (15.8%; 95% CI 3.4%‐39.6%) IOS procedures and in 7 (36.8%; 95% CI 16.3%‐61.6%) conventional impression procedures. This difference did not reach statistical significance (McNemar test, *P*=.13).

### Secondary Outcomes: Active Bedside Procedural Time

No statistically significant difference in active bedside procedural time was detected between the 2 procedures when all initiated procedures were analyzed. For IOS, the mean duration was 3.02 minutes (SD 0.72; median 3.2, IQR 2.4-3.4; range 1.5-4.2; n=19). For the conventional impression procedure, the mean recorded duration was 2.92 minutes (SD 0.78; median 3.2, IQR 3.0-3.3; range 0.0-3.5; n=19); the value of 0.0 minutes reflected the prematurely terminated impression attempt. The mean within-participant difference was 0.10 minutes (SD 1.15; median 0.1, IQR −0.8 to 0.71; range −1.5 to 3.2; sign test, 2-sided P>.99).

### Order Effects and Exploratory Analyses

The counterbalanced allocation sequence was used to reduce potential order-related effects. In exploratory analyses, no statistically detectable evidence of an order effect was observed across the assessed outcomes.

**Figure 4.**
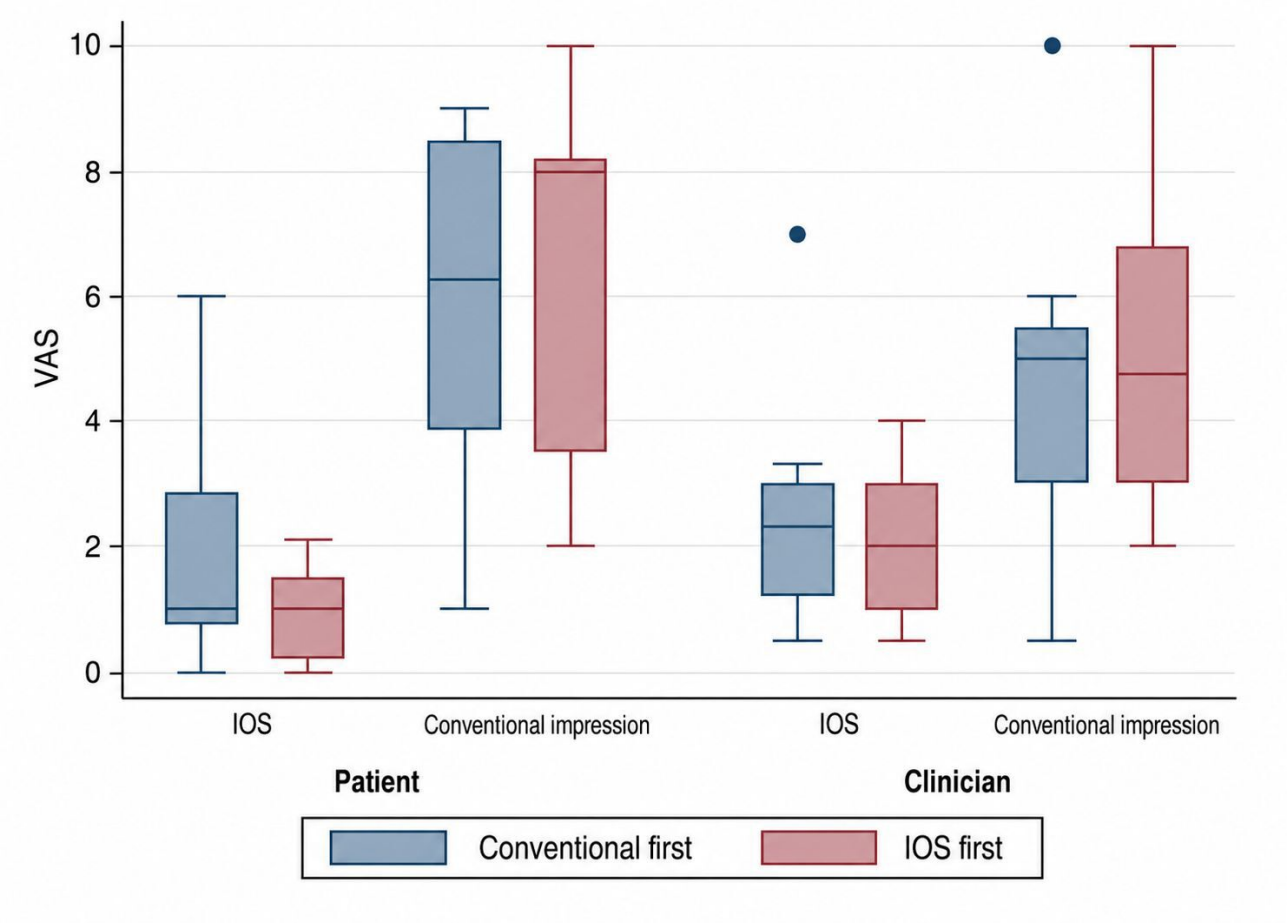
Visual analog scale (VAS) ratings of intraoral scanning (IOS) and conventional impressions stratified by the sequence of the procedure (conventional first vs IOS first). One participant VAS rating was missing; boxplots reflect only the available cases.

Participant-rated discomfort scores for IOS and conventional impression taking, stratified by procedural sequence, are shown in Figure 4. Between-group comparisons indicated no statistically significant differences according to whether IOS or conventional impression taking was performed first (participant VAS for IOS: *P*=.65; participant VAS for the conventional procedure: *P*=.82; rank-sum tests). One participant did not provide VAS ratings; therefore, these analyses were based on the 18 available cases.

Clinician-rated VAS scores also did not differ significantly according to procedural sequence (IOS: *P*=.54; conventional procedure: *P*=.74; rank-sum tests). Analyses of the paired differences between procedures, calculated as conventional impression minus IOS, likewise showed no statistically significant order effects for participant ratings (*P*=.13) or clinician ratings (*P*=.46).

For adverse reactions, associations with procedural sequence were not statistically significant (defensive movements during IOS: *P*=.58; defensive movements during the conventional procedure: *P*=.65; Fisher exact test). Given the small feasibility sample, these exploratory analyses cannot exclude clinically relevant order or carryover effects.

### Exploratory Analyses: Cognitive Status and Oral Health Markers

Cognitive status was not associated with participant-reported discomfort for IOS (Spearman ρ=−0.093; *P*=.71; n=18) or the conventional procedure (ρ=−0.030; *P*=.91; n=18). Cognitive status was also not significantly associated with oral health markers (MMSE vs DMFT: ρ=0.29, *P*=.23; MMSE vs number of teeth: ρ=−0.14, *P*=.58; n=19). Exploratory associations between participant discomfort and adverse reactions were not statistically significant for IOS (VAS vs gag reflex: *P*>.99; VAS vs defensive movements: *P*=.30).

## Discussion

### Principal Findings

This nonrandomized, counterbalanced, crossover feasibility study suggests that bedside maxillary IOS can be performed in hospitalized older adults living with dementia and is associated with a substantially lower immediate procedural burden than a conventional alginate impression.

Although both procedures were largely feasible, IOS was completed in all participants, whereas 1 conventional impression had to be terminated due to withdrawal of cooperation. Beyond feasibility, all burden-related outcomes consistently favored IOS: participants reported markedly lower discomfort, clinicians rated IOS as easier to manage, and the gag reflex occurred far less frequently during IOS. Active bedside procedural time was comparable between the 2 procedures when all initiated procedures were analyzed; however, this metric reflected patient-facing bedside time rather than the total digital or conventional workflow time.

Taken together, these findings indicate that the key advantage of IOS in dementia care is not feasibility alone, but the reduction of procedure-related burden under real-world bedside conditions.

Our results align with previous studies in general dental populations showing improved comfort and reduced gag reflex with IOS compared with conventional impressions [20,21] and extend these findings to a cognitively impaired inpatient population under real-world bedside conditions. This is particularly relevant in dementia care, where tolerance for sustained intraoral manipulation is often limited and distress may escalate rapidly [15]. Importantly, agreement between participant-rated discomfort and clinician-rated manageability was low, reflecting different but complementary perspectives. This finding is consistent with evidence from dementia research indicating that proxy assessments capture aspects of care feasibility and burden that are not fully aligned with patients’ subjective experience, yet remain clinically meaningful [36]. Considering both perspectives, therefore, strengthens the interpretation of procedural burden in this population.

The present findings should not be interpreted as evidence that IOS directly enables full prosthodontic rehabilitation or improves downstream treatment outcomes in people living with dementia. The data support a narrower conclusion: IOS reduced immediate burden during initial maxillary data acquisition in this small inpatient sample. Whether this translates into improved assessment, documentation, care planning, treatment completion, or implementation across care settings requires prospective multicenter studies. Our findings should be interpreted within a broader framework of responsible digital dentistry. The FDI Policy Statement emphasizes that digital dentistry applications should be critically evaluated and implemented with attention to data privacy, data quality, professional expertise, cost, accessibility, and equity [30]. These considerations are particularly relevant in dementia care, where digital tools should reduce rather than increase barriers to oral health care.

This perspective is supported by prior work showing that acceptance of routine dental procedures decreases with increasing care dependency [37]. By lowering procedural burden at the assessment stage, IOS may facilitate inclusion of people living with dementia in diagnostic workflows, interdisciplinary decision-making, and longitudinal monitoring. Recent evidence suggests that IOS-derived data may support remote triage and interprofessional collaboration in selected settings; however, whether such approaches are scalable in dementia care requires further evaluation [24].

This study has several limitations. First, the sample size was small, and the study was conducted at a single geriatric ward of a university medical center, limiting external validity. The findings cannot be directly generalized to nursing homes, outpatient dental practices, or settings with different staffing and workflow conditions. Second, the alternating allocation sequence was used for counterbalancing but did not constitute true randomization; selection bias and unmeasured sequence-related effects therefore cannot be excluded. Third, both procedures were performed during a single visit, and no fixed washout period was prespecified. This pragmatic approach was chosen to minimize repeated patient transfers, additional consent or assent procedures, and disruption of routine geriatric ward care in a frail inpatient dementia population. However, fatigue, stress, sensitization, or habituation after the first procedure may have affected participant behavior, discomfort ratings, and clinician-rated manageability during the second procedure. Although alternating sequence allocation and exploratory order analyses did not indicate statistically detectable order effects, the small sample size limits the ability to rule out clinically relevant carryover effects. Fourth, all procedures were performed by 1 highly experienced prosthodontist with expertise in geriatric dentistry and IOS. This ensured procedural consistency but may have overestimated feasibility and limits generalizability to operators with less experience or without dementia-sensitive training. Fifth, clinician-rated manageability was not blinded and may therefore be affected by operator expectations. Finally, procedure duration reflected active bedside procedural time rather than the total clinical workflow time. Scanner setup, case creation, calibration, data processing, and data saving were not included, which may underestimate total IOS workflow time in routine implementation.

In addition, implementation of IOS in dementia care requires adequate operator training and should not be understood as a substitute for clinical judgment or dementia-sensitive communication. As highlighted by the FDI Policy Statement, insufficient professional expertise may lead to overreliance on digital tools and poor decision-making, while rapid technological advances may increase costs and disparities in access [30].

### Conclusions

Bedside IOS was feasible in this small sample of hospitalized older adults living with dementia and was associated with lower immediate procedural burden than a conventional alginate impression procedure. Active bedside procedural time was comparable in the analysis of all initiated procedures, although total digital and conventional workflow times were not assessed. These findings support further multicenter studies evaluating IOS under routine dementia care conditions, including different operators, care settings, workflow integration, training requirements, and downstream patient-centered outcomes.

## Supplementary material

10.2196/92879 Multimedia Appendix 1 Consent to participate in the study and study information in easy-to-read language (translated from German into English).

## Abbreviations

CONSORT: Consolidated Standards of Reporting Trials
DMFT: decayed, missing, and filled teeth
ICC: intraclass correlation coefficient
IOS: intraoral scanning
MMSE: Mini-Mental State Examination
VAS: visual analog scale
