# Supplementary material for: Bedside Intraoral Scanning to Reduce Procedural Burden in Older Adults With Dementia: Nonrandomized Counterbalanced Crossover Feasibility Study
Source: JMIR Form Res. 2026 Aug 6;10:e92879. doi: 10.2196/92879 (PMC13446792; doi:10.2196/92879)
Supplement: Supplementary file 1 [file formative-v10-e92879-s001.pdf]

Consent to participate in the study in easy-to-read language (Translated from German into English)

Study information in easy-to-read language (Translated from German into English)

Consent to Take Part in the Study

Sometimes there is a red bar next to the text.
The red bar means: examples.

Sometimes there is a blue bar next to the text.

The blue bar means: explanations of words

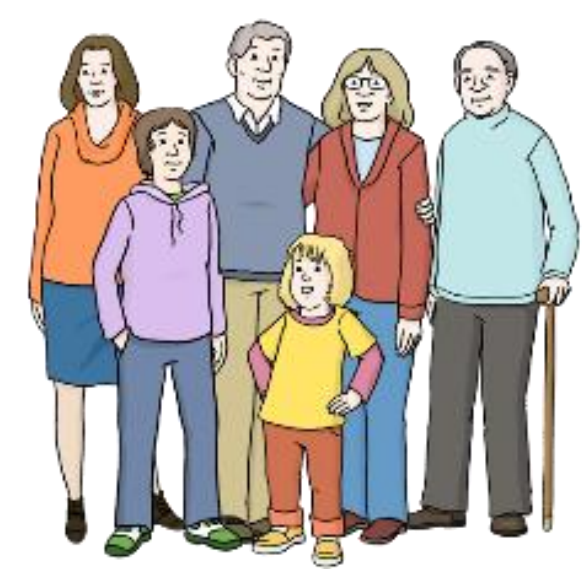

We use the male form in this text,

but we mean all people.

Consent means:

You agree to something.

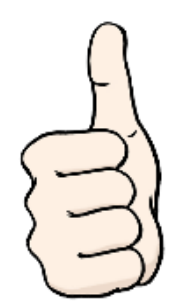

In a study, doctors and researchers examine people.

They need people who want to help them.

They study, for example, which treatment works best.

Another word for study is research.

The study is called (in difficult language):

Comparison of the practicality of digital and conventional impression techniques in people with dementia-related reduced treatment capability.

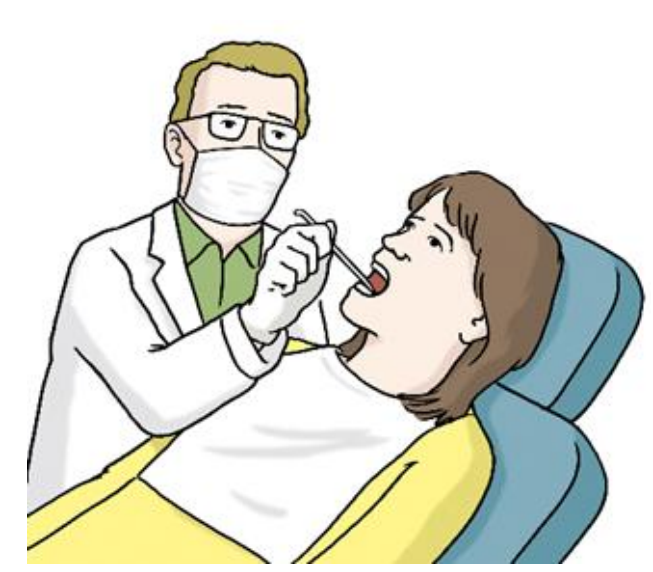

This means:

The dentists want to find out:

Can people with dementia have their teeth taken as impressions?

For this, the teeth are recorded once with a camera,
and once taken with impression material (like soft putty).

You want to take part in this study.

You have talked with ___________________________
about the study.

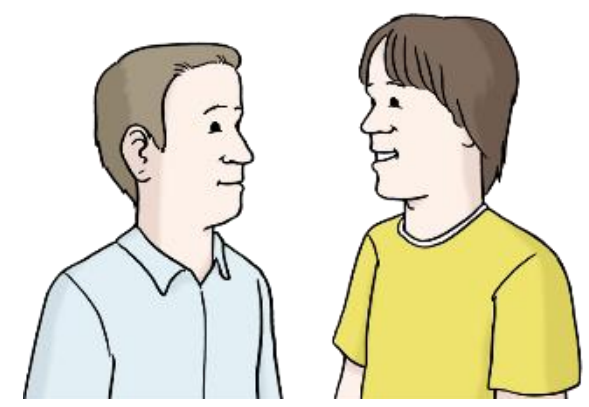

You have received all information about the study:

Why is this study being done?

What risks are there in this study?

What are your rights and duties?

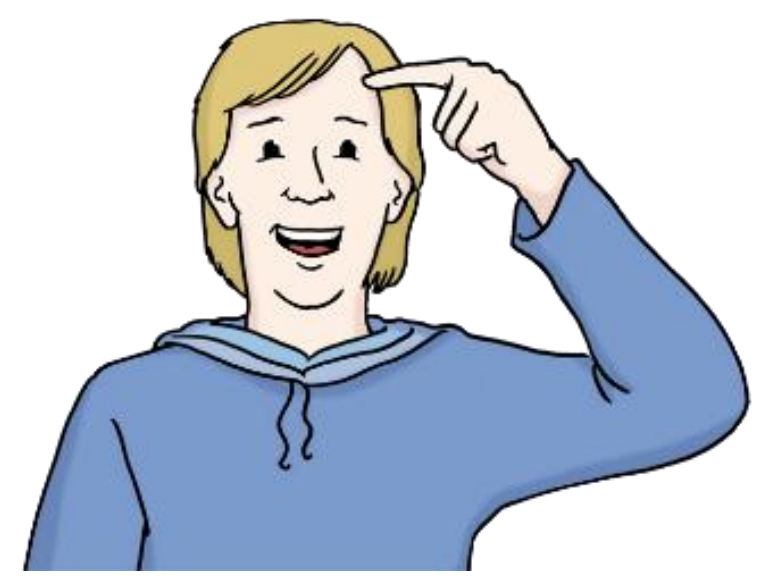

You could also read all the information.

You have received copies for your records:

Information about the study

Your consent form

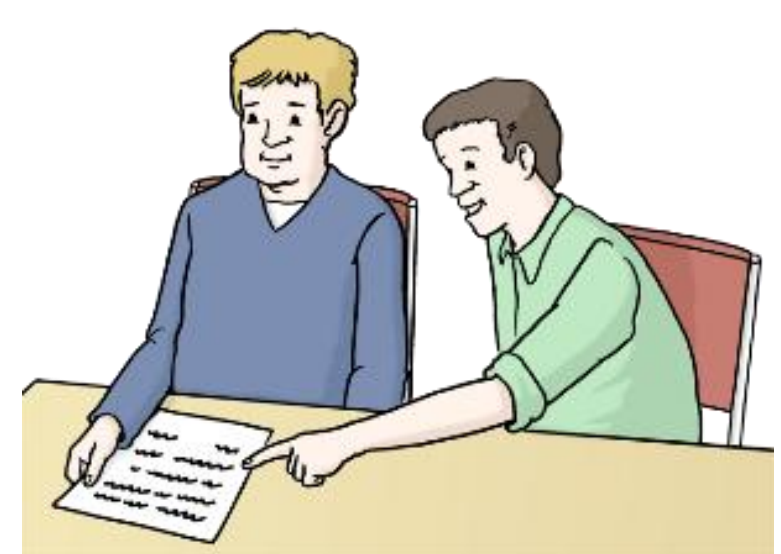

The dentist has answered all your questions.
You have talked about these things:

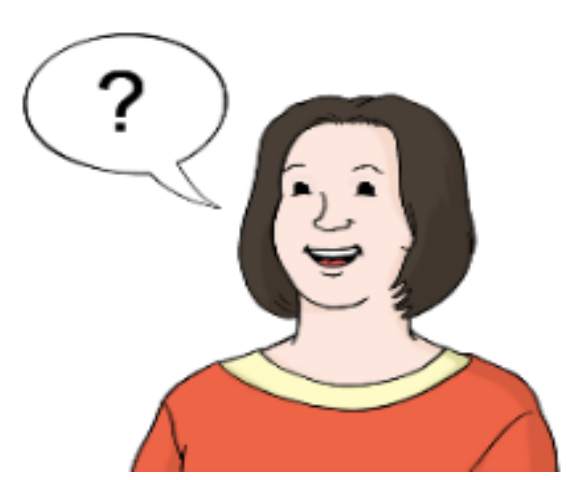

____

____

Your participation is voluntary.

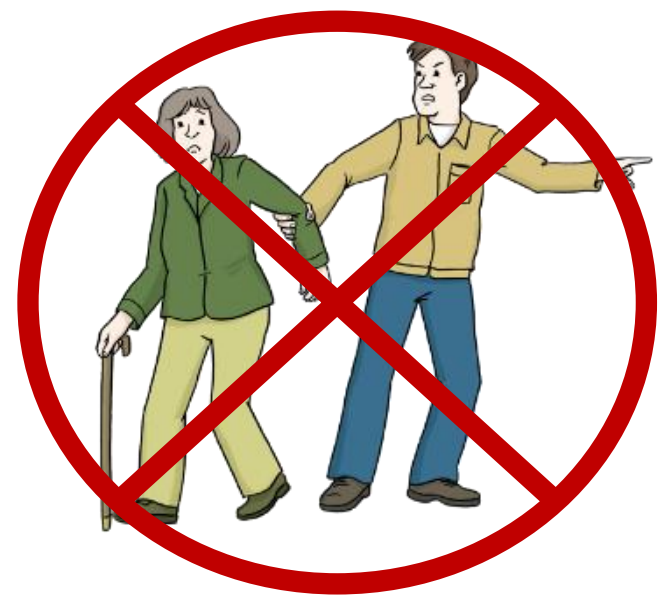

> This means:
>
> You take part in the study because you want to.
>
> No one is forcing you.
>
> You agree to take part.

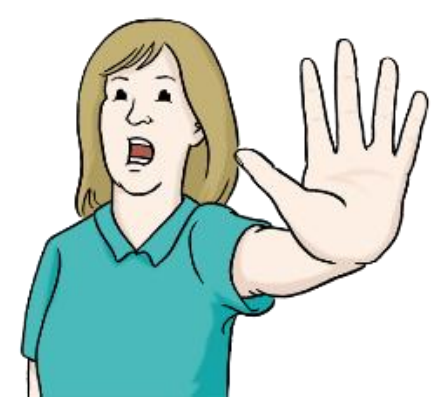

You can always say:

You do not want to take part anymore.

You do not have to say why you want to stop.

If you stop, you will not be disadvantaged:

not in your treatment

not with your dentist

You agree to take part in the study

Name:

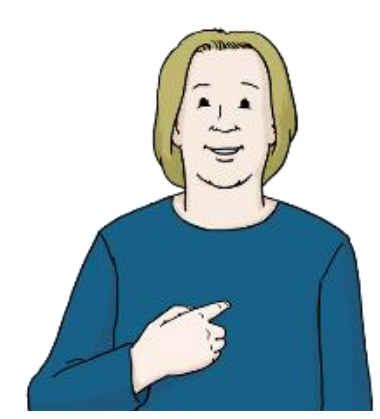

Place and date:

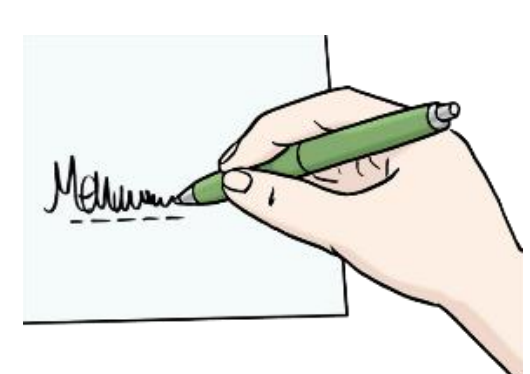

Your signature:

You have talked with this dentist:

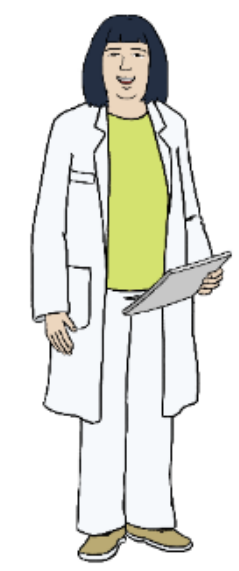

Name:

Place and date:

Signature of the dentist:

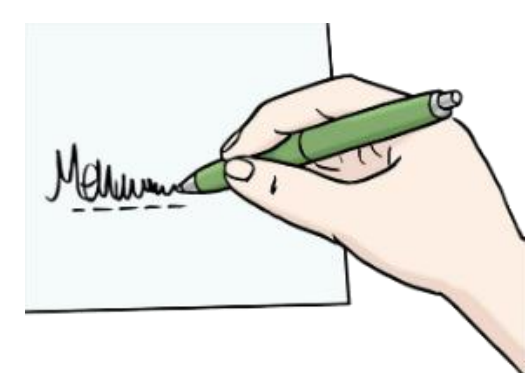

Consent for the Use of Your Data

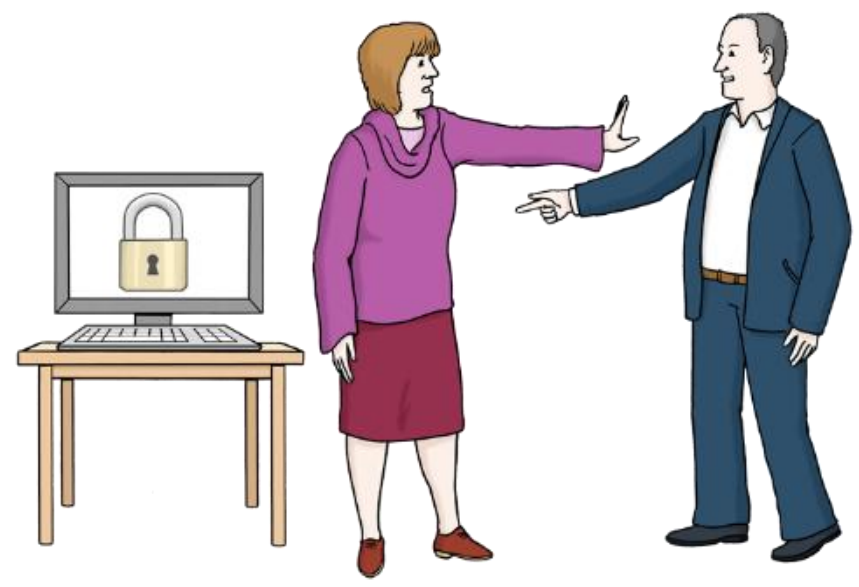

This part is about data protection.

Data protection means:

You decide

- who may use your data, and
- for what purpose your data may be used.

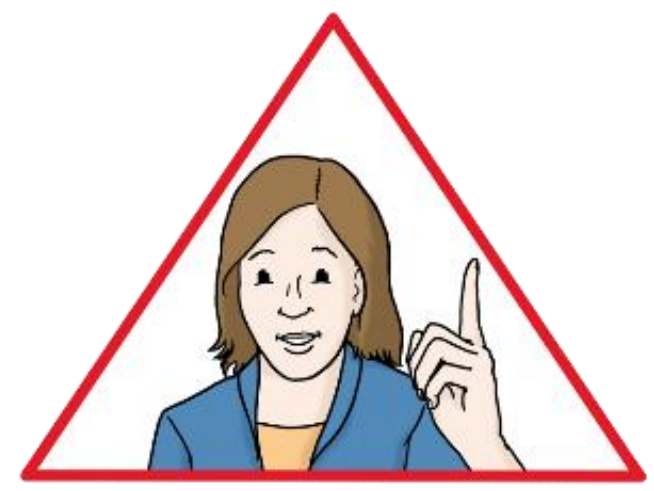

Not everyone may know your data.

Not everyone may use your data.

Not everyone may share your data with others.

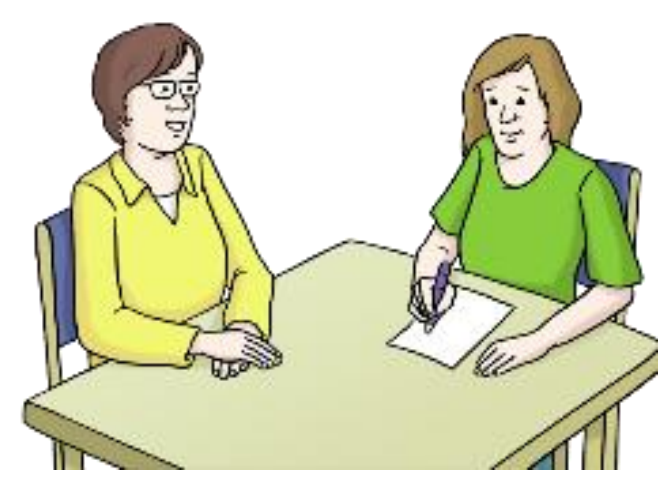

Because you want to take part in this study,

you have to give us some personal data.

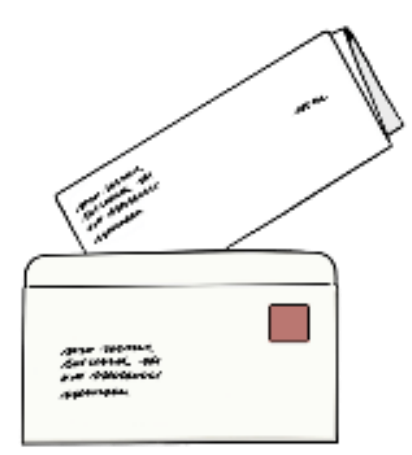

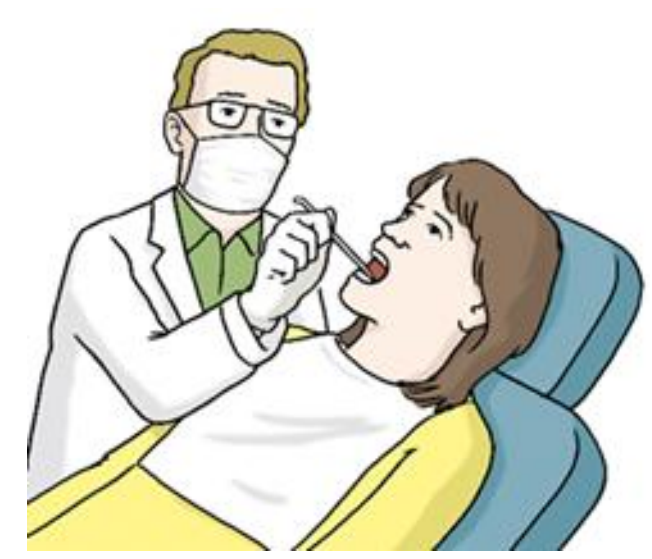

Personal data means, for example:

- your name
- your address
- your date of birth
- how many teeth you have

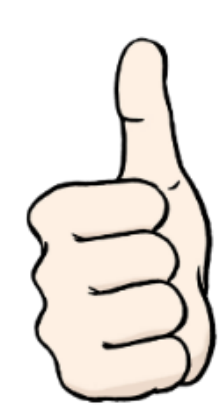

The dentists need your data for this study.

For that, they need your consent.

You agree that your data can be used for this study.

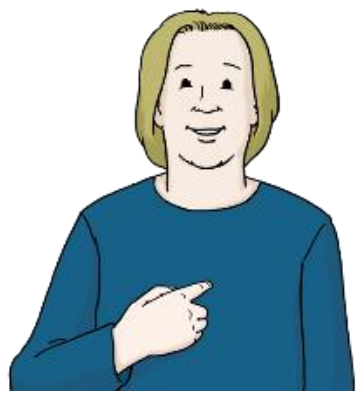

Name:

____

Place and date:

____

Your signature:

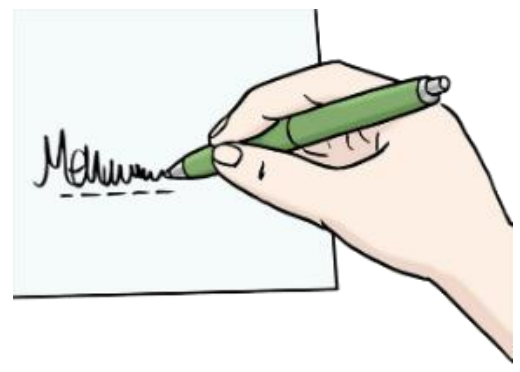

____

You have talked with this dentist:

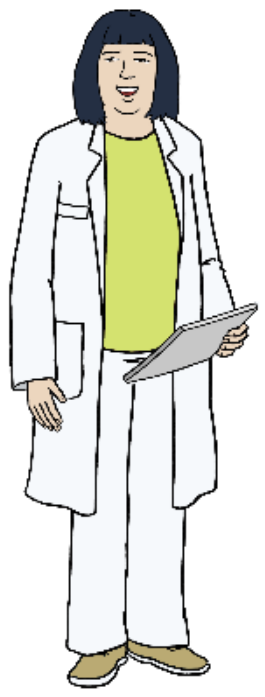

Name:

____

Place and date:

____

Signature of the dentist:

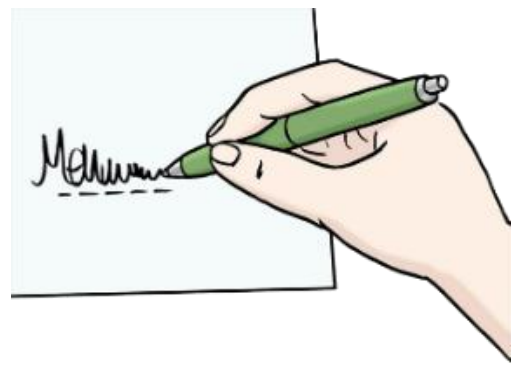

____

Study Information in Easy Language

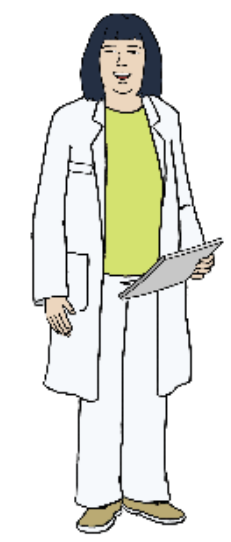

Sometimes there is a red bar next to the text.
It shows examples.

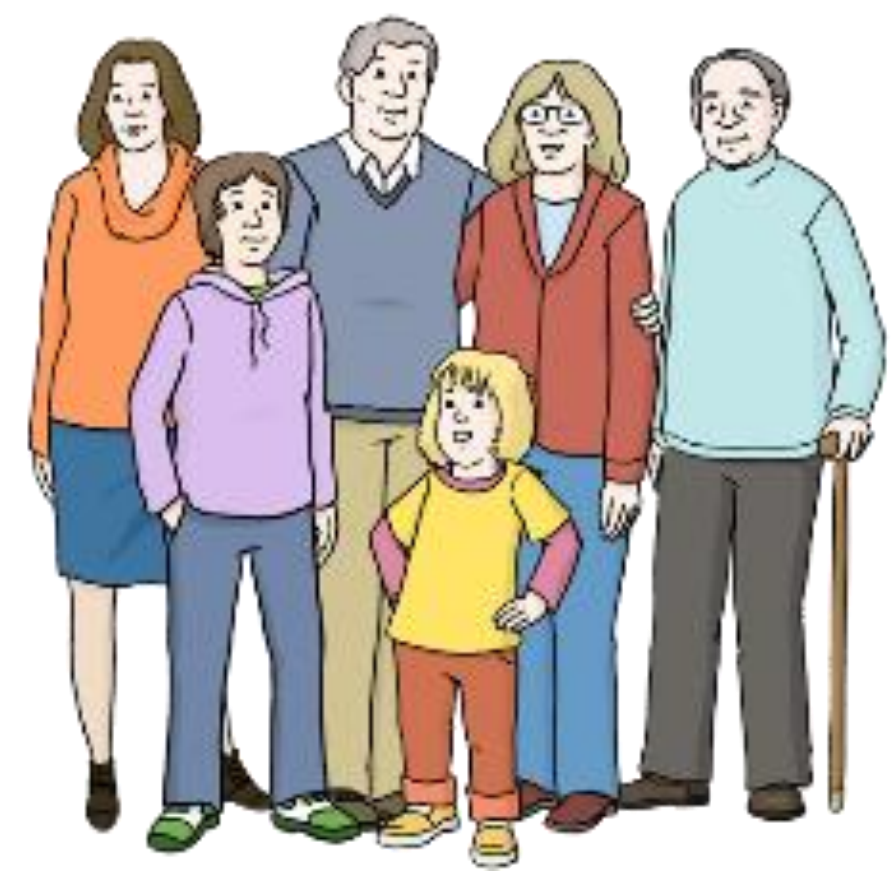

Sometimes there is a blue bar next to the text.
It shows word explanations.

We use the male form in this text,
but we mean all people.

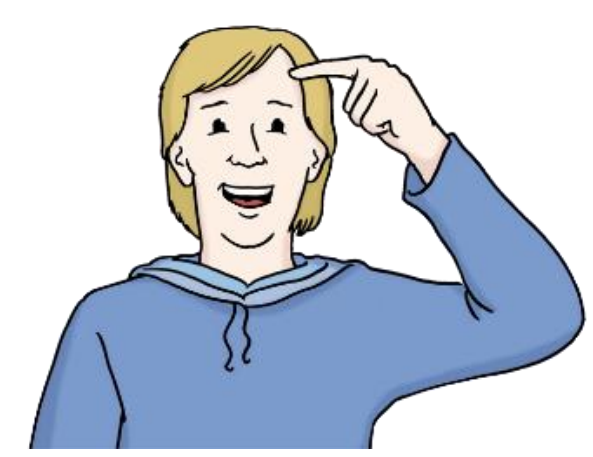

You want to take part in a study.
This text gives you information about the study.

Study means:
Doctors and researchers examine people.
They need people who want to help them.
They look at which treatment helps best.
Another word for study is research.

Read this text carefully.
Also talk to your dentist about the study.

If you have questions,
the dentist will answer them.

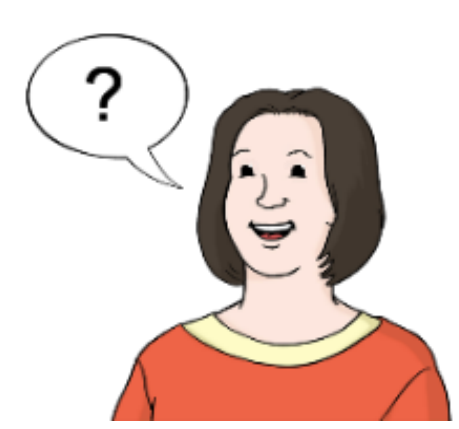

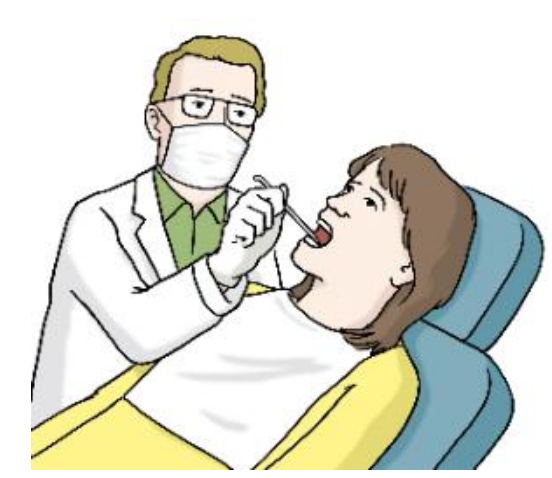

The study

The study is called (in difficult language):

Comparison of the practicality of digital and conventional impression techniques in people with dementia-related reduced treatment capability

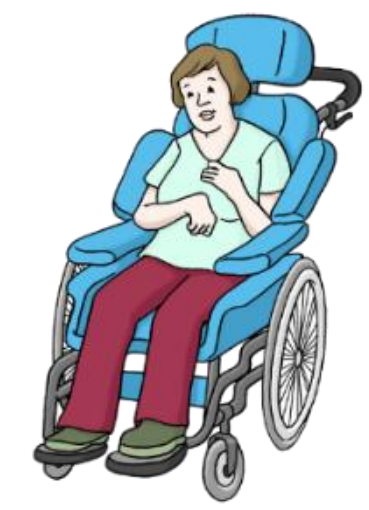

This means:

Can people with dementia have their teeth taken as impressions?

The dentists want to know:

- How well does the camera work to scan the teeth?
- How well does the soft impression material work?
- How do the participants feel about the impressions?
- How does the dentist feel about the impressions?

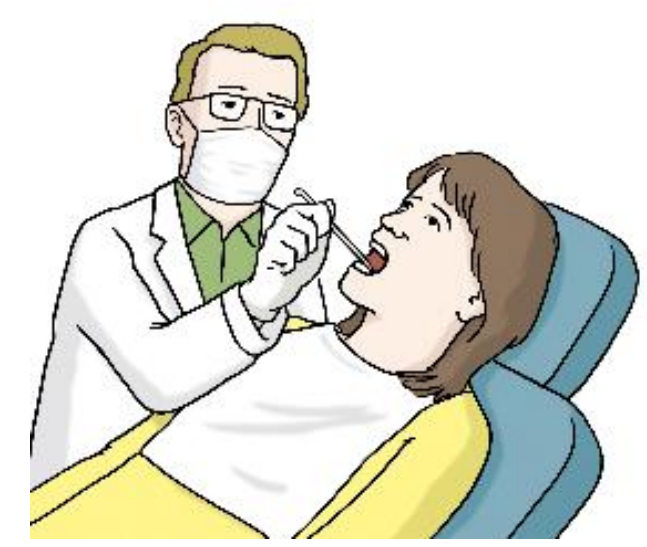

Each participant will have two impressions:

One with a camera and one with soft material.

The teeth are filmed once with a camera

and moulded once with modelling clay.

After each moulding, the study participant says how they liked the moulding.

After the mouldings, the dentist says how they liked the moulding.

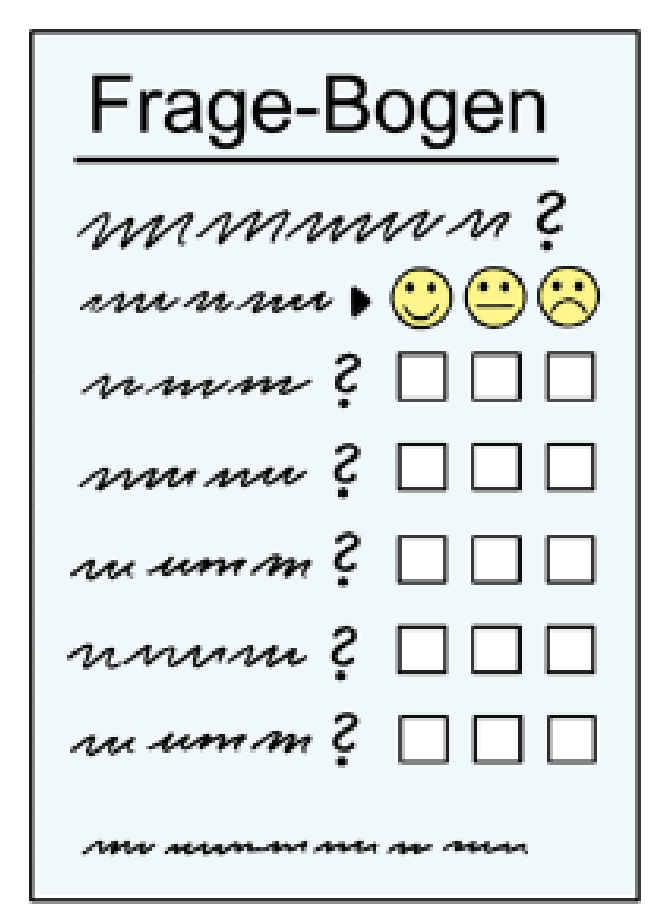

Costs

The study is free of charge for you.

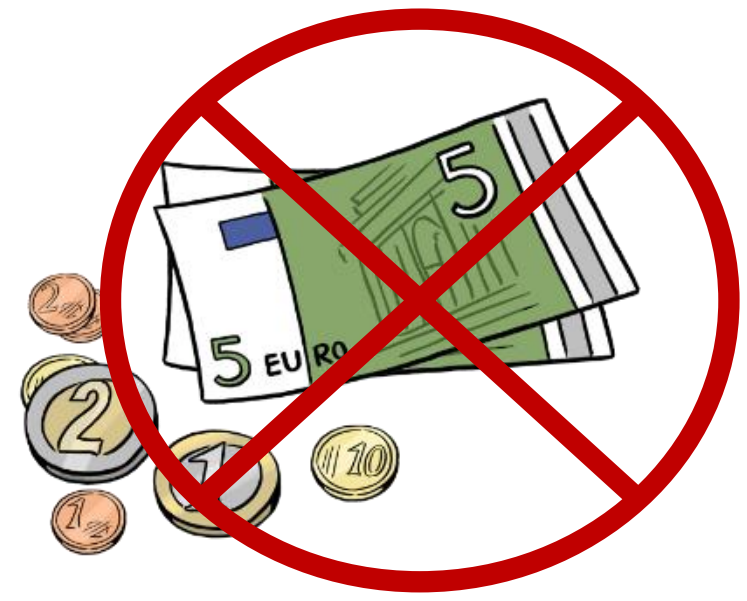

Stopping the study

Your participation is voluntary.

You take part because you want to.

No one forces you.

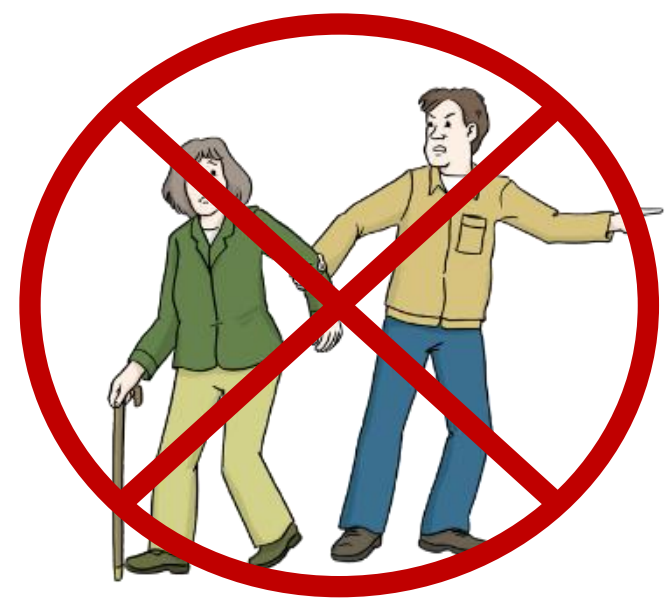

> Das bedeutet:
> Sie machen bei der Studie mit.
> Keiner zwingt sie dazu.
> Sie sind mit der Studie einverstanden.

You can always say:

You don’t want to take part anymore.

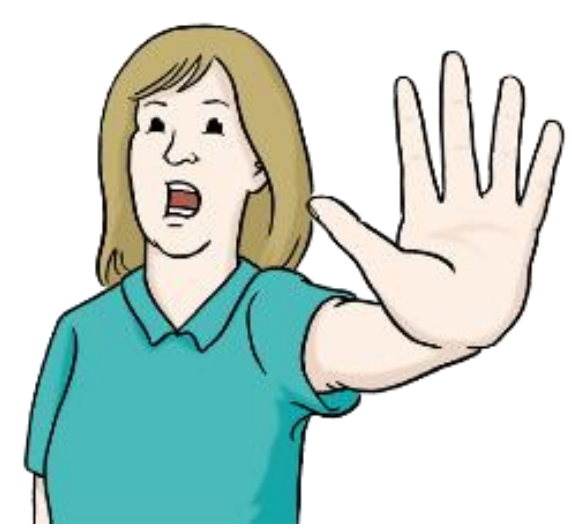

You don’t have to say why.

If you stop taking part,

you will not have any disadvantages.

Many people take part in the study.

Together, the results can help

other people with dementia in the future.

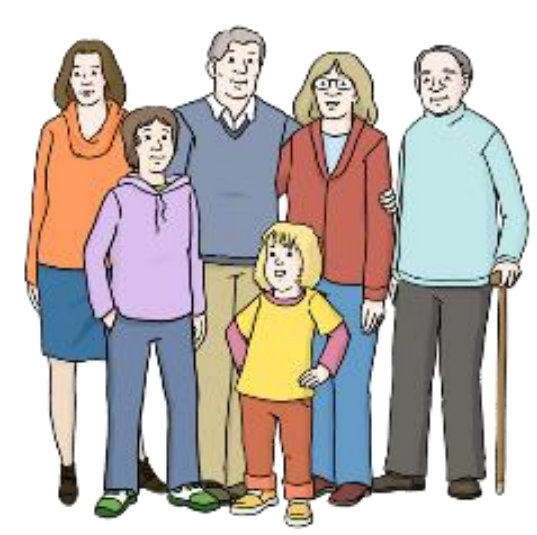

Data Protection

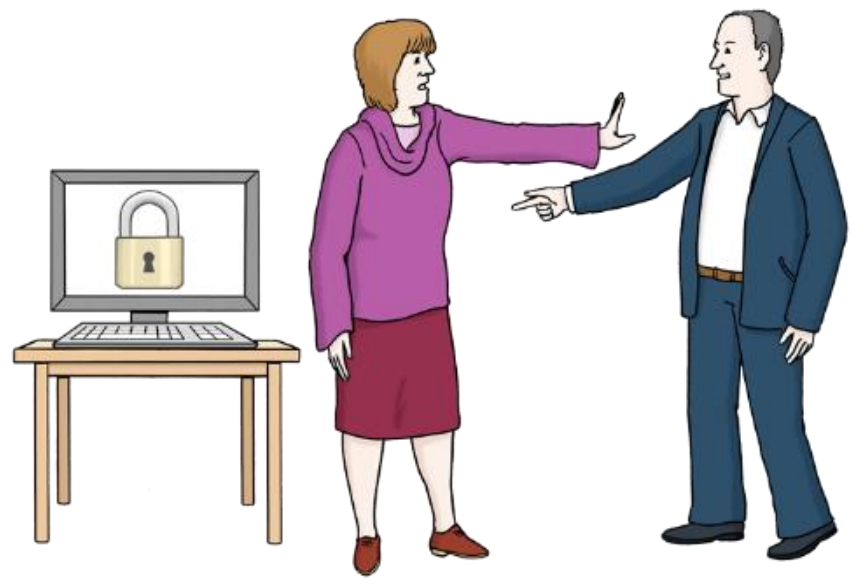

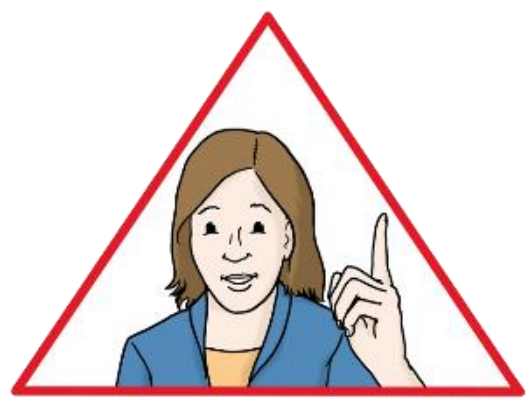

Data protection means:

You decide

- who may use your data, and
- for what purpose your data may be used.

Not everyone may know or use your data,

and not everyone may share it with others.

There is a law for data protection.

It is called the General Data Protection Regulation (GDPR).

In German: Datenschutz-Grundverordnung (DSGVO)

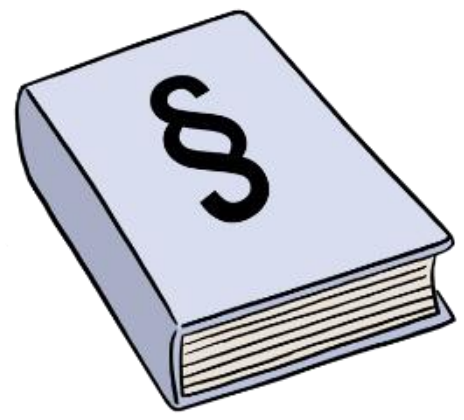

Because you take part in this study,

we need to use some of your data.

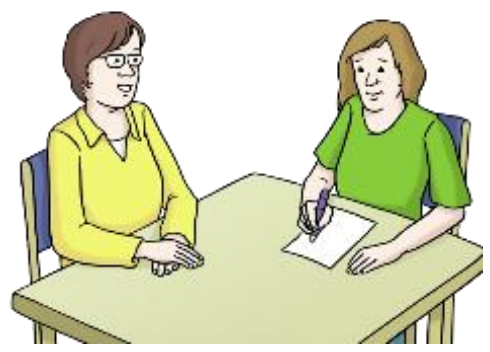

Everyone working on the study must keep your data **confidential.**

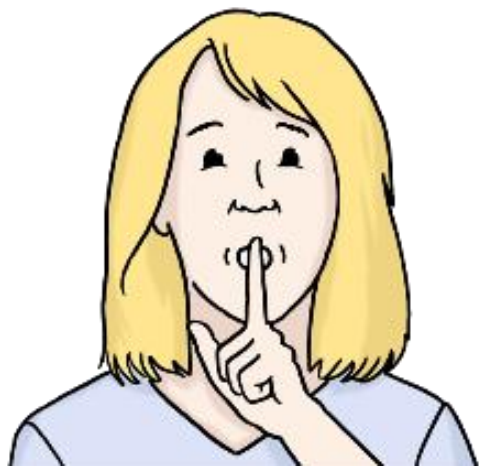

Confidential means:

No one may tell your data to others.

No one may give your data to others.

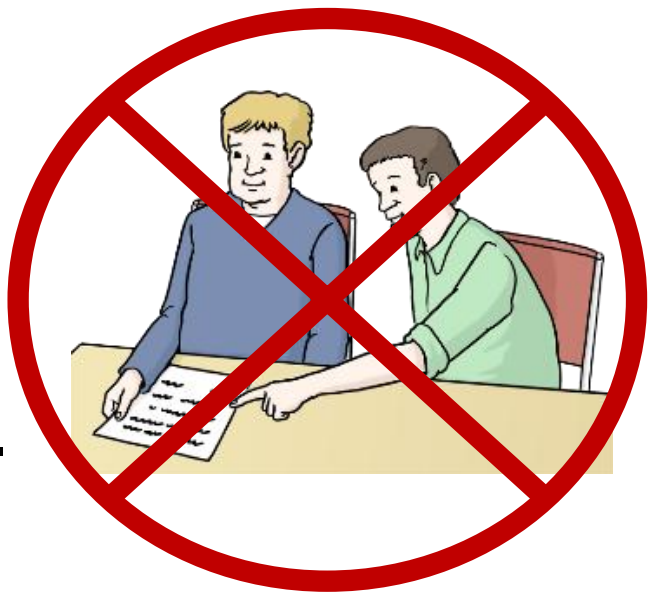

The dentists may use your personal data only for this study.

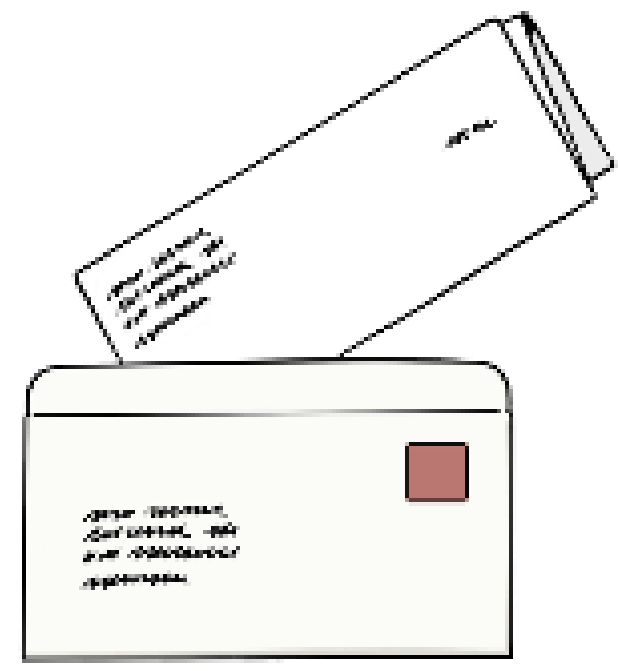

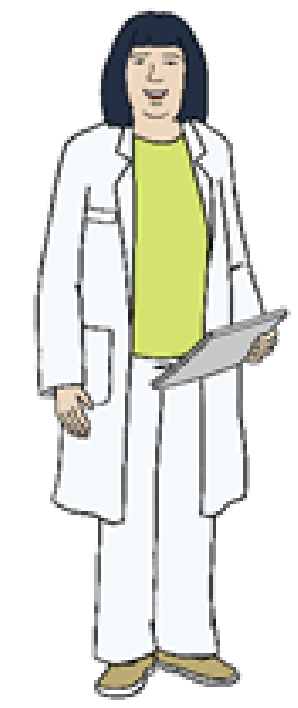

Personal data means, for example:

your name

your address

your date of birth

how many teeth you have

For that, the dentists need your consent.

Consent means:

You agree to something.

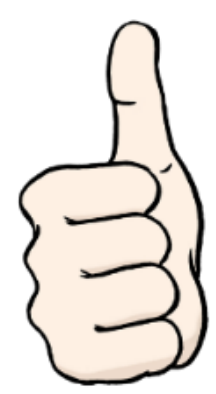

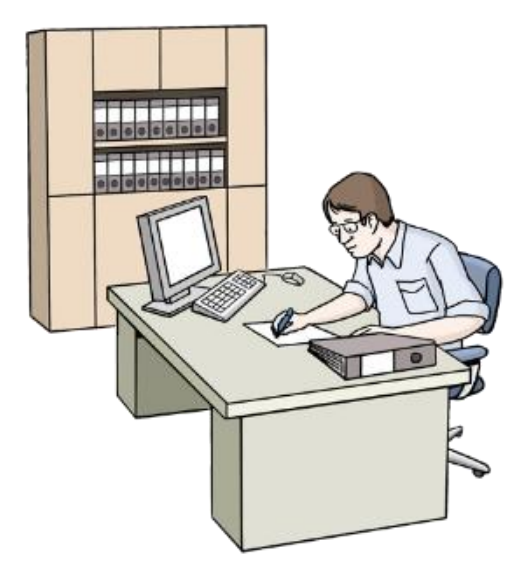

The dentists will

- store your data only for this study, and
- use your data only in this study.

<u>Pseudonymisation</u>

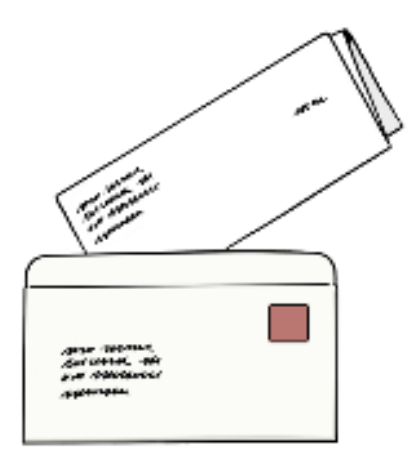

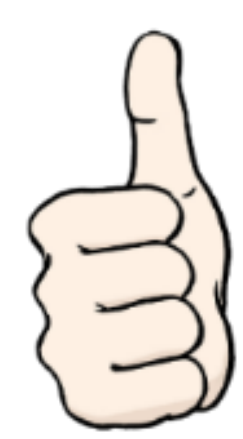

We store your data with your consent.

We replace your personal information with a code.

In difficult language, this is called pseudonymisation.

This means:

No one can see to whom the data belong

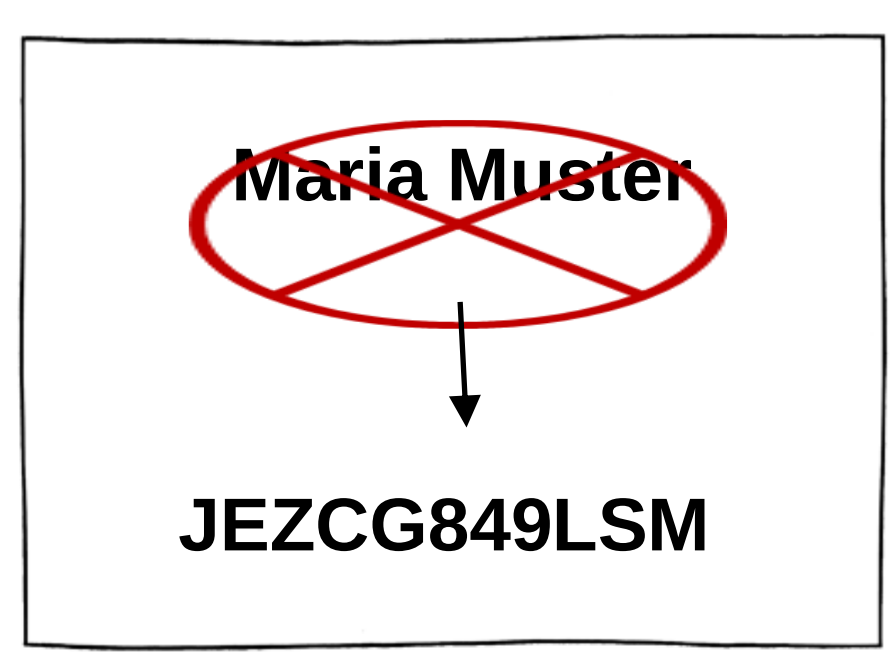

Anonymisation

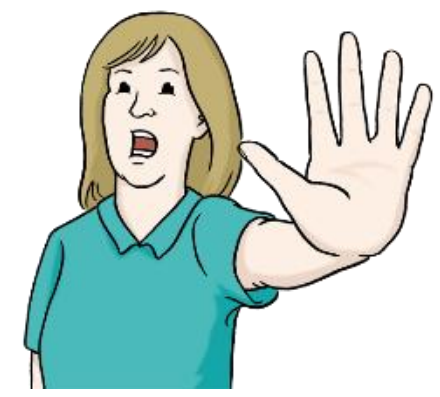

If you decide to stop taking part in the study,

we will delete your personal data.

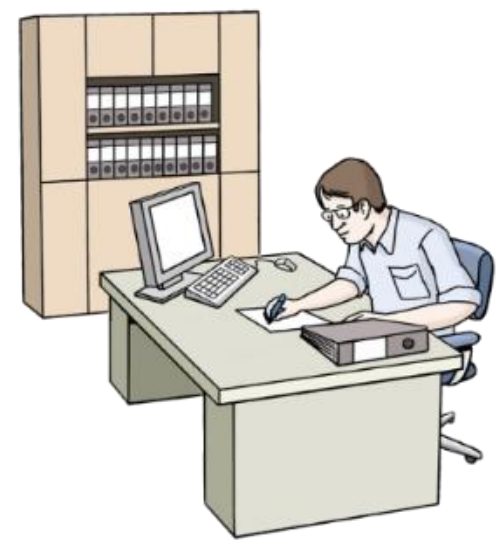

The study results will still be used —

but they will be anonymised.

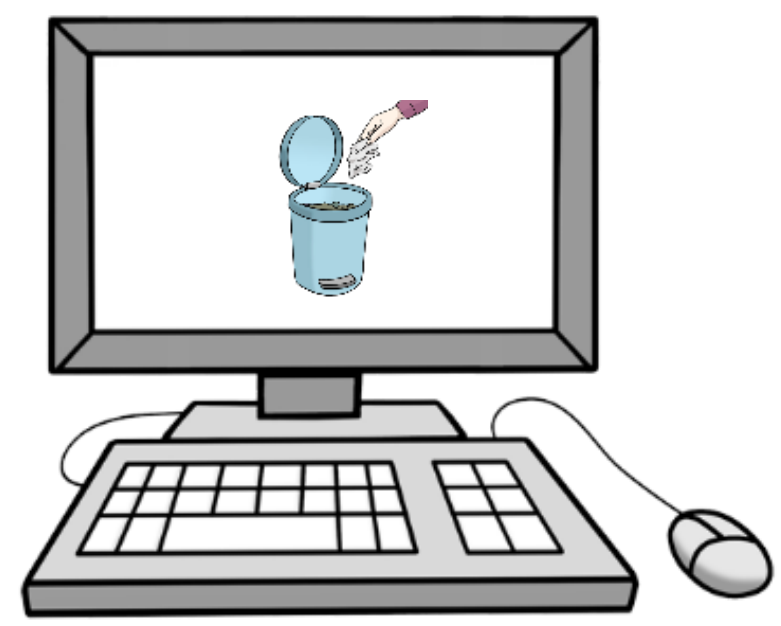

Anonymisation means:

We have personal data about you.

This tells us:

These results are yours.

We delete your personal data.

This includes, for example,

your name and date of birth.
